# Supplementary figures and images for: Interfacial mechanisms for stability of surfactant-laden films
Source: PLoS One. 2017 May 17;12(5):e0175753. doi: 10.1371/journal.pone.0175753 (PMC5436193; doi:10.1371/journal.pone.0175753)

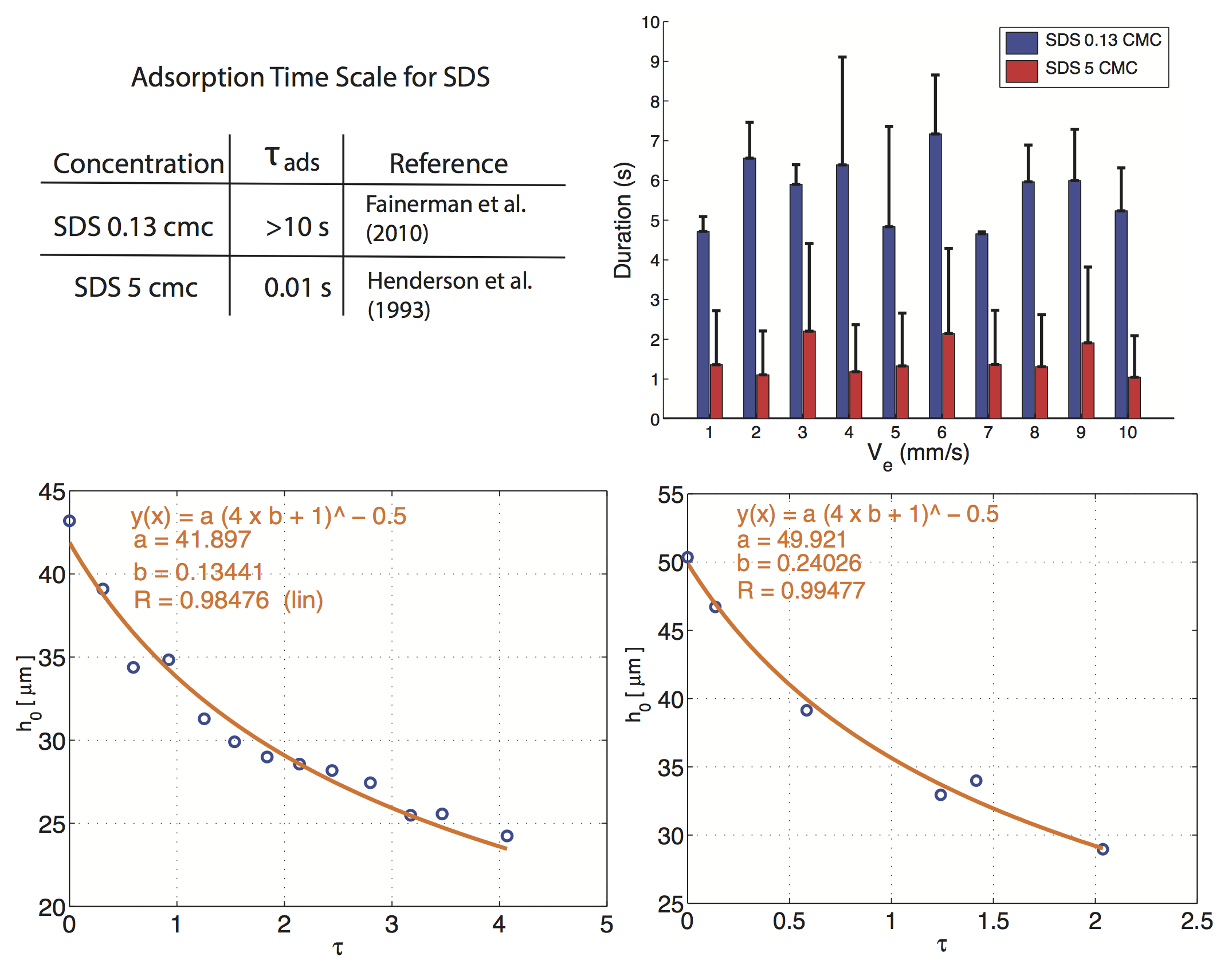

Supplement: S1 Fig — (A) Adsorption time scales for SDS at 0.13 cmc and 5 cmc. (B) Duration of drainage for SDS films at above and below cmc. (C & D) Representative drainage results of below and above cmc of SDS at 1 mm s−1, with b corresponding to our fitting parameter α. (TIFF) [file pone.0175753.s001.tiff]

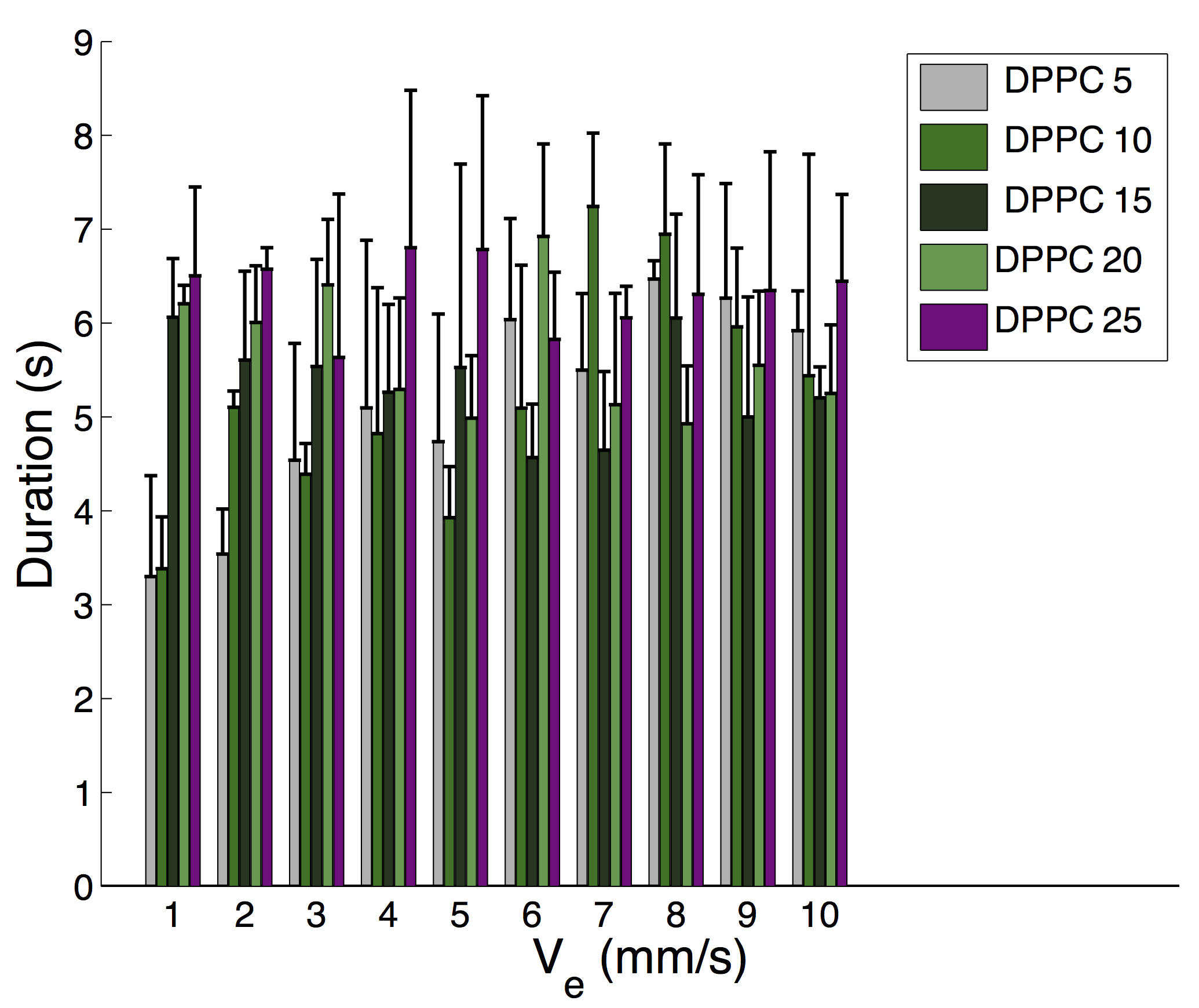

Supplement: S2 Fig — Raw-data showing duration of drainage (in seconds) for DPPC films at different surface pressures and elevation velocities. (TIFF) [file pone.0175753.s002.tiff]
